# Effective Subcritical Butane Extraction of Bifenthrin Residue in Black Tea

**DOI:** 10.3390/molecules22040560

**Published:** 2017-03-30

**Authors:** Yating Zhang, Lingbiao Gu, Fei Wang, Lingjun Kong, Guangyong Qin

**Affiliations:** 1 Henan Provincial Key Laboratory of Ion Beam Bio-engineering, and School of Physics and Engineering, Zhengzhou University, 100 Science Avenue, Zhengzhou 450001, China; liuxiaoli@zzu.edu.cn (Y.Z.); gulingbiao@foxmail.com (L.G.); wsh@zzu.edu.cn (L.K.); 2 School of Chemistry and Molecular Engineering, Zhengzhou University, 100 Science Avenue, Zhengzhou 450001, China; panpeipei@zzu.edu.cn

**Keywords:** subcritical butane, pesticide residue, bifenthrin, black tea

## Abstract

As a natural and healthy beverage, tea is widely enjoyed; however, the pesticide residues in tea leaves affect the quality and food safety. To develop a highly selective and efficient method for the facile removal of pesticide residues, the subcritical butane extraction (SBE) technique was employed, and three variables involving temperature, time and extraction cycles were studied. The optimum SBE conditions were found to be as follows: extraction temperature 45 °C, extraction time 30 min, number of extraction cycles 1, and in such a condition that the extraction efficiency reached as high as 92%. Further, the catechins, theanine, caffeine and aroma components, which determine the quality of the tea, fluctuated after SBE treatment. Compared with the uncrushed leaves, pesticide residues can more easily be removed from crushed leaves, and the practical extraction efficiency was 97%. These results indicate that SBE is a useful method to efficiently remove the bifenthrin, and as appearance is not relevant in the production process, tea leaves should first be crushed and then extracted in order that residual pesticides are thoroughly removed.

## 1. Introduction

Tea is of great economic importance [1], not only as the most widely-consumed beverage worldwide, but tea extracts are also used as flavoring in sweets and bakery products throughout the world [2]. According to our understanding, tea plant growth needs high humidity, found in tropical and subtropical climates [3], which renders it susceptible to damage by pests and fungus, resulting in large amounts of pesticides occurring [4]. Therefore, the resulting food-safety problem becomes a profound issue that must be solved over time [5]. In order to control the occurrence of pesticides, the establishment of a rapid and efficient way to remove pesticide residues in tea has great realistic significance for ensuring the safety of tea products, and for improving consumers’ health.

Several studies on pesticide removal from a variety of plants, including vegetable, fruits and crops, have been reported [6,7,8]. As a common and effective way to remove water-soluble pesticides, washing with water or soaking in salt solution has been used [9]. Further, studies have also verified that eluting with some chemicals (such as chlorine and ozone [10], hydrogen peroxide [11], hydroxy peracetic acid and detergents [12,13]), is highly effective in reducing the level of pesticides, even hydrophobic pesticide Additionally, hydrostatic pressure [14], biological and electrochemical oxidation [15], UV photolysis and TiO_2_ catalytic treatment [16], and ultrasonic processing [17,18] have already been intensely studied. However, there still exist some limitations to these techniques, because of chemical compositions of the pesticide residue, or the characteristic of material. For example, the quality of tea will be seriously affected if it is treated by techniques which depend on water, or solution containing water [19]. It is worth noting that secondary pollutants might be produced by some treatment. Other than the above methods, supercritical fluid extraction (SFE) and accelerated solvent extraction (ASE) are used to quickly detect levels of organochlorine and organophosphorus pesticides on tea leaves, without accessional pollutants and water participant [20,21,22]. However, when applying SFE and AS, a certain degree of damage to plant samples can be generated in its grinding pre-treatment. To complicate matters, both techniques require a high working pressure within the range of 7~50 MPa [23,24].

Subcritical fluid extraction is a relatively simple, rapid and environmentally friendly technology, which is mostly used in extraction of flavors [25], special functional edible oils [26], natural pigments [27], and medicinal compositions [28]. When compared to other techniques, it has the advantage of a lower operating temperature and pressure, shorter extraction time, simpler operational process and environmental compatibility [29,30]. N-butane is one of the most common subcritical fluids, owing to lower critical pressures and temperatures, and excellent dissolving power [31]. In particular, it leaves no residue in the product and has the merit of a low boiling point and cheap price [32]. Therefore, subcritical butane extraction (SBE) may be an ideal approach when applied to large-scale industrial application so far. Nevertheless, application of SBE technology for pesticide removal from plants has seldom been reported.

China is one of the main producers of tea, but, unfortunately, the export of tea has been declining gradually, and one of the main causes is pesticide residues [33]. However, there are few efficient ways for removing pesticide residues in tea so far. Bifenthrin, a pyrethroid insecticide, has the characteristic of a broad pesticidal spectrum, thus it is widely-used in controlling pests of tea growth [34]. Black tea is a fermented tea, acting as a leading product. In this study, we used this tea with residual bifenthrin as the experimental material, then the SBE parameters (temperature, time and extraction cycles) were optimized for the removal of bifenthrin residue, based on response surface methodology (RSM) with a three-variable-three-level Box-Behnken design (BBD). Subsequently, HPLC and GC-MS analyzed the chemical substances which impacted tea taste and aroma. Overall, this project aimed at developing a highly efficient and simple operational approach for the removal of pesticide residues from tea products, while at the same time, maintaining the tea’s quality.

## 2. Results

### 2.1. Optimization of Subcritical Fluid Extraction Condition

Subcritical butane extraction parameters were optimized by the response surface methodology (RSM), according to the Box-Behnken design (BBD). Based on the results of the single-factor experiments, the range and center point values of three independent variables, including (A) temperature, (B) time, and (C) number of extraction cycles, were determined. Table 1 exhibits the response values (bifenthrin extract content, *Y*) for different experimental combinations. The results suggested that the extraction efficiency of bifenthrin is significantly changed depending on the extraction conditions (Table 1).

The multiple regression analysis was employed based on the experimental data, and the predicted response *Y* to bifenthrin extract content can be obtained by the second-order regression equation (in terms of coded factors): *Y* = 2.88 + 0.88 × A + 0.076 × B − 0.013 × C − 0.08 × A × B − 0.46 × A × C + 0.15 × B × C + 0.46 × A^2^ + 0.18 × B^2^ + 0.0005 × C^2^. To ensure the reliability of the model, the determination coefficient (*R*^2^) was calculated and came to 0.9859, which indicated an agreement between the experimental and predicted values. The analysis of variance was shown in Table 2. It showed that the lack of fit was insignificant (*p* > 0.05), suggesting that the model equation was adequate for predicting the bifenthrin extract content in any combination of the variables’ values. In total, the ANOVA demonstrated the Fisher’s F-test for the model had a high *F*-value (54.53), and a quite low *p*-value (<0.0001), so the model was significant.

**Figure 1:**
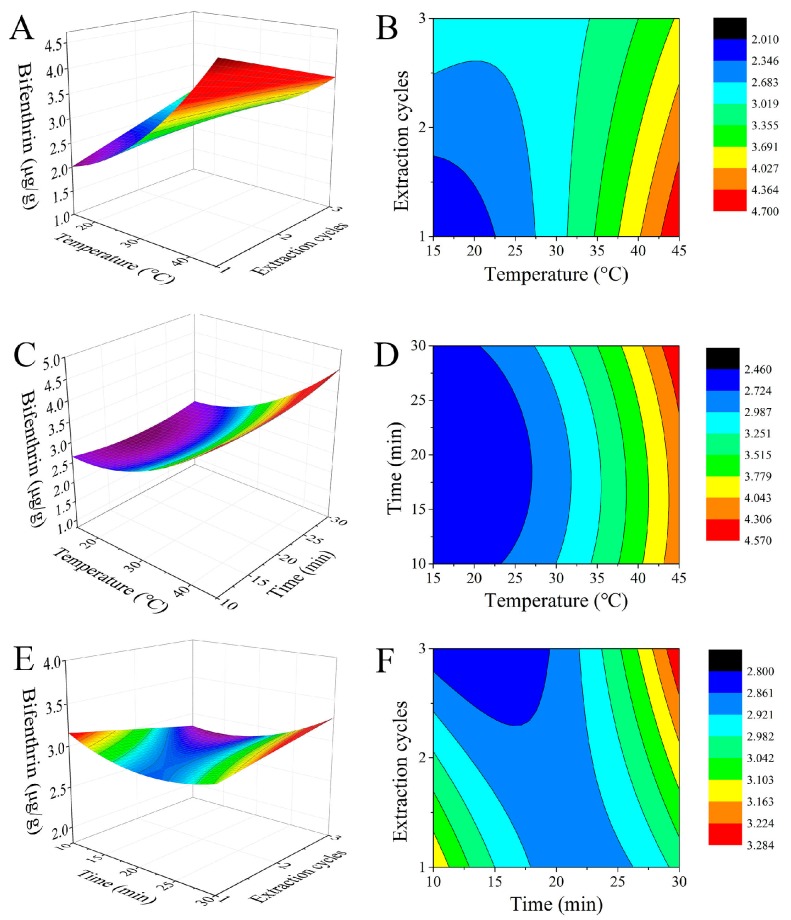
Response surface plots (**A**, **C** and **E**) and contour plots (**B**, **D** and **F**) of bifenthrin extract content affected by temperature, extract time and extraction cycles.

The ANOVA of the linear, quadratic and interaction terms of the model are shown in Table 2 as well. A (extraction temperature), A^2^ (the quadratic of extraction temperature) and AC (the interaction of extraction temperature and the number of extraction cycles) were quite significant (*p* < 0.01), and B^2^ (the quadratic of extraction time) was significant (*p* < 0.05). Therefore, we can make such a conclusion: the impact factor of extract content of bifenthrin had a scope trend from extraction temperature (important), extraction time, to the number of extraction cycles (negligible). The three-dimensional (3D) response surface and two-dimensional (2D) contour plots based on the regression equation model are shown in Figure 1. The response value increased steadily at the designed range of temperature from 15 to 45 °C among a number of extraction cycles and the extraction time. In addition, when the number of extraction cycles increases, the extraction efficiency could be effectively enhanced under the same temperature. Three variables (including A, B and C) existed extremum in Figure 1, so derivative operation of the model equation was performed. The results showed that when A, B and C was 45 °C, 1 and 27.85 min respectively, the extraction efficiency increased to the maximum (~94%). Based on the theoretical prediction, the optimal extraction conditions were chosen as extraction temperature 45 °C, number of extraction cycles 1, and extraction time 30 min. The three replication experiments under the same condition proved that the average extraction efficiency reached 92%, close to the theoretical prediction. To this end, the regression equation is reliable for the analysis and prediction of pesticide residues’ elimination by butane.

### 2.2. The Effect of Crushing of Tea Leaves on Bifenthrin Removal

**Figure 2:**
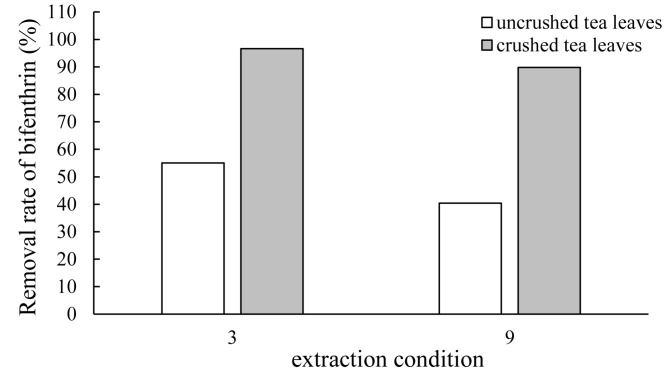
The extraction efficiency of bifenthrin of uncrushed tea leaves and crushed tea leaves. The numbers 3 and 9 in the horizontal axis represent the extraction condition numbered 3 and 9 in Table 1, respectively.

The crushed tea leaves can be used in instant tea and food additives. To make further enhancements in the extraction efficiency of bifenthrin, we studied the effect of pulverization on the extraction efficiency. Crushed ones (average size of tea particles is 0.3 mm) were divided into two groups and extracted on the basis of the conditions 3 and 9 in Figure 2, respectively. The results manifested that the extraction efficiency of bifenthrin in the crushed sample increased to 97% and while 90% under the extraction condition 3 and 9, which increased separately 42% and 49%, compared with the uncrushed ones, indicating pesticide residues can be more easily removed from crushed tea leaves. Thus, if the appearance is not important, tea leaves should be crushed firstly and then extracted in order to remove the pesticide residues thoroughly.

### 2.3. Content Changes of Catechins, Theanine and Caffeine in Tea Leaves after Subcritical Butane Extraction

Catechins, theanine and caffeine are the main substances which determine the flavor and color of tea. We detected them in the control sample and treated one in the assigned condition (extraction temperature 45 °C, number of extraction cycles 1, extraction time 30 min) via HPLC, aiming at the determination of the quality changes of tea, before and after the experiment. In chromatographic analysis, calibration curves were constructed by plotting peak area (*Y*) versus the corresponding concentration (*x*, g/g), and the correlation coefficients (*R*^2^) of all calibration curves were greater than 0.9921, which indicated that these were sufficiently reliable for the analysis of such substances (Table 3). Based on a linear regression equation obtained from calibration curves, the six kinds of catechins, theanine and caffeine content in the control and treated samples were calculated. In the six kinds of catechins, their loss rate ranged from 2.86% to 6.64%, except for catechin (14.58%). Further, the theanine was 4.54% and the caffeine had the lowest loss rate, at 1.37%. Overall, the subcritical butane extraction method had a minor effect on catechins, theanine and caffeine in the tea leaves, that is, the technology influenced the quality of tea slightly.

### 2.4. Aroma Components in Tea Leaves after SBE Processing

**Figure 3:**
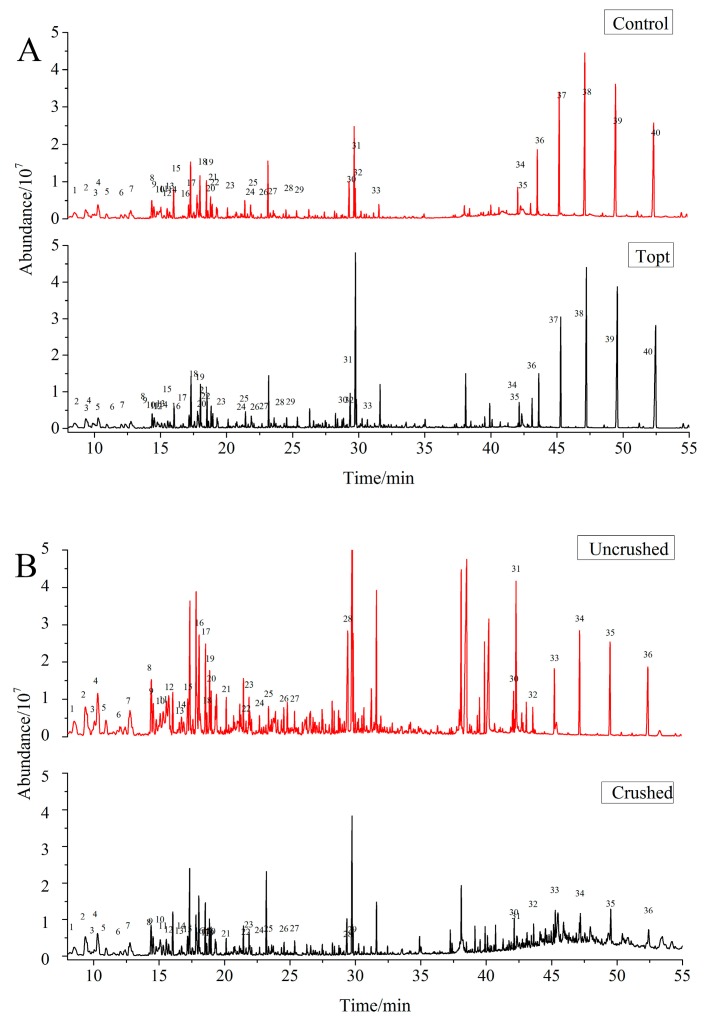
Total ion chromatograms of aroma composition of the intact and crushed tea leaves after subcritical butane processing. **A**: the intact tea leaves, including the control sample and Topt sample (the treated sample), which was treated by the SBE processing under the optimum condition: extraction temperature 45 °C, extraction time 30 min, number of extraction cycles 1. **B**: the crushed and uncrushed tea leaves were processed by SBE under the condition numbered 3. The number in the figure represents the number for every substance.

The characteristic aroma is also an important criterion for the evaluation of the tea quality. In this study, the flavor profiles of the control and treated samples were firstly obtained by GC-MS chromatography (Figure 3A), and relative contents of the components were determined by area normalization. In all, 40 major volatile compounds were identified and grouped into several classes, (including 8 alcohols, 6 alkanes, 5 ketones, 10 aldehydes, 3 esters, 1 terpenes and 7 other compounds (Table S1), showing that the SBE had no impact on the aroma components. The five main characteristic aroma components were further screened to analyze their relative content changes. Among them, the amount of linalool had no change, and there was a relatively small variation for linaloloxide, nerol and 2-(*E*)-hexenal, however, the gradient of relative content for β-ionone reached to 64.49% (Table 4).

The flavor profiles of the crushed sample after SBE processing were also obtained by GC-MS chromatography (Figure 3B). 36 major volatile compounds were identified and grouped in classes consisting of 8 alcohols, 6 alkanes, 5 ketones, 8 aldehydes, 3 esters, 1 terpene, and 6 other compounds (Table S2). The results showed that the SBE also had no impact on the kinds of aroma components in the crushed tea leaves, while the relative contents of the five main characteristic aroma components all decreased significantly, by approximately 46.44–60.06% (Table 4).

## 3 Discussion

### 3.1. SBE Is a More Efficient Method to Reduce Pesticide Residues

Subcritical fluid extraction is widely-used to extract the flavors, functional edible oils, natural pigments and medicinal composition [25,27]. This technology employed lower temperature, lessened pressure and nontoxic organic solvent, thus making it convenient for large-scale industrial application, and we firstly attempted to study it to remove pesticide residues. Three key parameters for SBE, including temperature, time and extraction cycles, were explored and analyzed. Given that high temperatures negatively affect the taste of tea, we set the upper limit of extraction temperature at 45 °C. The extraction temperature had the most impact on bifenthrin extract content, and the extraction efficiency of bifenthrin increased with increasing temperature. This phenomenon can be interpreted as the vapor pressure of the solute in subcritical butane mixtures enlarged with the increasing temperature from 15 °C to 45 °C, leading to high solubility [35,36] and extraction efficiency of bifenthrin. The extraction cycles had the smallest impact, which indicated that bifenthrin could dissolve rapidly and completely in butane. Finally, the optimum condition of SBE was 45 °C, =30 min, extraction cycles 1. In such a case, the extraction efficiency was about 92%. Notably, the crushed leaves even reached to 97%. By way of comparison, the reduction of chlorpyrifos pesticide in cherry tomatoes by high hydrostatic pressure treatment was demonstrated, and the maximum extraction efficiency reached 75% [37]. Pesticide residue removal from vegetables by ozonation reached 77% [38], and the effectiveness of chlorine dioxide to remove pesticide residues on fresh lettuce was 40–80% [39,40] Therefore, SBE is a much more efficient method to wipe out pesticide residues than others.

### 3.2. SBE Had Very Little Effect on Parts of Tea Taste

Although the SBE could efficiently remove pesticide residues, the quality of tea after SBE treatment must be evaluated if the SBE is considered for use in large-scale processing. The taste of tea, an important factor in tea quality, was affected significantly by the catechins, theanine and caffeine. Our results indicated that the content of catechins, theanine and caffeine had little loss after SBE processing, which was likely because the hydrophilic and lipophilic structure of these substances resulted in low dissolubility in butane [36]. Additionally, the shape, color and taste of tea remained the same after SBE.

### 3.3. SBE Had Some Effect on Parts of the Aroma Components

Aroma is another crucial factor ascribed to the tea quality. In this study, aroma components of tea leaves are monotone decline after SBE processing. The loss rate of relative content for β-ionone, a main characteristic aroma component, reached 67% (Table 4). Compared with intact tea leaves, the loss rates were much more significant (Table 4). This can be explained by parts of the aroma substances being hydrophobic, leading to slightly higher dissolubility in butane. When the tea was ground, tea cells were destroyed and fat-soluble aroma components were easily carried away by butane because the aroma components adhere to the surface of leaves. However, the content loss of the aroma substances caused by SBE processing could be compensated by adding flavors to tea products. Overall, the content of aroma components can be reduced by SBE, but this problem can be solved to ensure tea quality by flavoring measures. Therefore, SBE can be regarded as a highly-efficiency method for removing residual pesticides.

## 4. Materials and Methods

### 4.1. Sample

The tea leaves were picked from one plantation (Xinyang, China). In experimental processes, the bifenthrin concentration (100 g/L, Shanghai Raw Agricultural Biochemical Co., Ltd., Shanghai, China, in July 2014) was adjusted to twice the suggested value. Three days after spraying the pesticide, tea leaves at the top were picked and processed into black tea, then transferred to a laboratory within the same day, at a temperature of 4 °C.

### 4.2. Chemicals and Instrument

The butane was purchased from Puyang Petrochemical Limited Company (Puyang, China), and the purity was higher than 99%. Bifenthrin (≥95%), phenethyl acetate (≥99%) and triphenyl phosphate (TPP) (≥95%) were used as internal standard and purchased from Dr. Ehrenstorfer Company (Augsburg, Germany). Acetonitrile, methanol, acetone, methylbenzene, ethyl acetate, butane, hexane, etc. were of chromatographic grade and purchased from J. T. Baker (Phillipsburg, NJ, USA). Anhydrous magnesium sulfate, sodium chloride, sodium citrate dibasic sesquihydrate, and citric acid was of an analytical grade and purchased from North Reagent Company (Tianjin, China). Primary secondary amine (PSA) sorbent was purchased from Supelco (Bellefonte, PA, USA). Six kinds of catechins, theanine and caffeine were purchased from Aladdin Company in Shanghai (≥95%). Other reagents were of analytical grade (North Reagent Company, Tianjin, China). The Chinese medicine pulverizer was manufactured in Shanghai Dianjiu by traditional Chinese Medicine Machinery Manufacturing Limited Company (Shanghai, China).

### 4.3. Sample Processing by Subcritical Butane Extraction (SBE)

**Figure 4:**
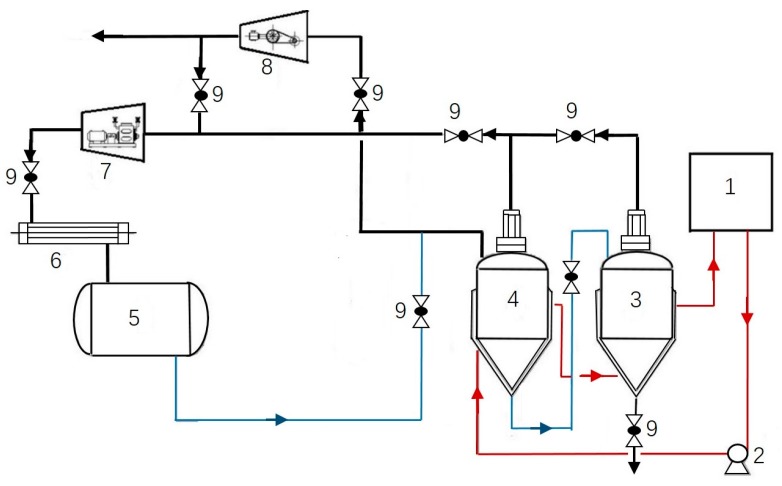
Schematic diagram of the apparatus used in subcritical butane extraction. 1: hot water tank; 2: hot water pump; 3: evaporation tank; 4: extraction tank; 5: solvent tank; 6: condenser; 7: compressor; 8: vacuum pump; 9: spherical valve.

The tea leaves with bifenthrin were treated by the SBE method, and the schematic diagram of the adopted apparatus (CBE-5L, Engineering Research Center for Subcritical Extraction Equipment, China) appears in Figure 4. 100 g tea leaves were placed into a filter bag to keep an intact shape, then put into the extraction tank, which was subsequently filled with 1 L butane, and was processed at a certain temperature and time under a typical pressure. After processing, the extraction solution in the extraction tank was injected into the evaporation tank. The solvent butane was vaporized and separated via the vacuum distillation. The vaporized butane became liquid by the compressor and condenser to be recycled. Finally, extracts containing bifenthrin residues were collected at the outlet of the evaporation tank, and the processed sample was taken out of the extraction tank.

### 4.4. Determination of Bifenthrin Level in the Processed Samples Using GC-MS/MS

#### 4.4.1. Bifenthrin Standard Curves

The standard stock solution (10 μg/mL) and internal solution (20 μg/mL) were prepared by dissolving bifenthrin and TPP in acetonitrile, respectively. The intermediate standard solutions were obtained by serial dilution of standard stock solutions with acetonitrile, with 100 μL TPP in every one as an internal standard. The final concentration of bifenthrin in the above solutions was 2, 5, 10, 20, 50, 100, and 200 μg/mL. Then, GC-MS/MS (Trace GC Ultra/TSQ Quantum GC, Thermo Fisher Scientific, Sunnyvale, CA, USA) analysis was performed on each solution, and the ratio (*Y*) of the bifenthrin peak area to the TPP peak area was plotted against the concentration (*X*) of bifenthrin. Linear regression analysis revealed a standard curve with the following equation: *Y* = 0.00347065 + 0.00188399*X (*R*^2^ = 0.9998).

#### 4.4.2. Sample Preparation

To analyze bifenthrin levels in the samples extracted by SBE method and the control, all samples were extracted according to five major steps (1) crushed tea leaves (2.0 g) were mixed with 10 mL ultrapure water in a 50 mL centrifuge tube, which was then soaked for 10 min; (2) 100 µL internal standard solution TPP (20.0 mg/L), 10 mL acetonitrile and 5 mL methylbenzene were added into the centrifuge tube and shaken for 1 min at 2000 rpm, and was then stored at 4 °C for 10 min; (3) A salting-out reagent kit was put into mixed solution, and was then immediately vibrated for 2 min at 2000 rpm on the vortex mix and centrifuged for 3 min at 9000 rpm; (4) An extract liquid of 1.5 mL was acquired and placed into a 2 mL centrifuge tube, containing 150 mg anhydrous magnesium sulfate, 40 mg PSA adsorbent, and 40 mg C18E adsorbent. The extract liquid was continuously vibrated for 2 min at 2000 rpm and centrifuged for 3 min at 9000 rpm; (5) The extraction solution (1.0 mL) was filtered through a 0.22 µm nylon filter prior to the GC-MS/MS analysis. All tests were conducted in triplicates.

#### 4.4.3. GC-MS/MS Analysis

In this study, a trace GC ultra with TR-pesticide column (30 m × 0.25 mm × 0.25 µm) was applied in the GC-MS/MS system. The carrier gas was helium, with a constant flow rate of 1 mL per minute. The injector temperature was set as 250 °C. The oven temperature was firstly arranged at isothermal 50 °C for 1 min, and increased to 150 °C at a rate of 25 °C/min, then sequentially raised to 260 °C at 5 °C/min, finally increased to 280 °C at 10 °C/min, and remained isothermal at 280 °C for 20 min.

### 4.5. Determination of Aroma Components Using GC-MS/MS

#### 4.5.1. Sample Preparation

Simultaneous distillation-extraction (SDE) was used for extracting the aroma components from tea [36]. Before the extraction, 10 g of crushed tea was placed into a 1000 mL round-bottomed flask (sample flask), which was coupled to one arm of the SDE apparatus, containing 200 mL boiling deionized water and 10 µL of phenethyl acetate as an internal standard (10 mg/mL). A 100 mL round-bottomed flask (solvent flask), containing 40 mL of tetrachloromethane as the extraction solvent, was attached to the other arm of the SDE apparatus. The sample flask was heated to a particular temperature, ensuring the slight boiling of the solvent, and the other flask was heated and maintained at 55 °C in a water bath. Then, the extraction process lasted for approximately 2 h. After dehydration by anhydrous sodium sulfate, the extraction solution was concentrated to 2 mL using a rotary evaporator and filtered through a 0.22 µm nylon filter prior to GC-MS analysis.

#### 4.5.2. GC-MS Analysis

The GC-MS system used in this study was Agilent 7890-5790, with DB-5MS column (30 m × 0.25 mm × 0.25 µm). In the typical system, helium was the carrier gas, with a constant flow rate of 1 mL/min. The injector and ion source temperatures were 200 °C and 250 °C, respectively. The oven temperature was isothermal at 40 °C for 2 min, then increased to 260 °C at 10 °C/min, and remained at 260 °C for 20 min. The mass spectrometer conditions were as follows: ionization mode, EI; electron energy, 70 eV; ion source temperature, 250 °C; quadrupole temperature, 150 °C; mass scan range, 33–500 amu; and solvent delay, 3 min.

### 4.6. Determination of Catechins, Theanine, Caffeine Using HPLC

Six kinds of catechins, theanine, caffeine contents were determined by HPLC following the ISO 14502-2:2005, ISO/DIS 19563, ISO 10727:2002, respectively. Fenugreek seed was purchased from a regional pharmacy, located in Zhengzhou, China. Samples were ground into powder with a Wiley Mill (Thomas Scientific, Philadelphia, PA, USA) and passed through different sieve sizes according to experimental design. The seed powder was air-dried for 24 h at 80 °C, and then stored at 4 °C for further use. Butane was purchased from Puyang Longyu Chemical Co., Ltd. (Puyang, China). Other chemicals and solvents used for this study were of either analytical or chromatographic grade, and all purchased from either Fisher Scientific Chemical (Loughborough, UK), or Sigma Aldrich (Steinheim, Germany).

## 5. Conclusions

SBE technology was firstly applied to remove bifenthrin pesticide residues from tea leaves, and different SBE conditions were surveyed. The optimum SBE condition was as follows: extraction temperature 45 °C, extraction time 30 min, number of extraction cycles 1, and the extraction efficiency reached 92%. Furthermore, the extraction efficiency increased to 97% if the tea leaves were grounded. Meanwhile, the results indicated the quality of tea fluctuated little after SBE treatment, especially for intact samples. Thus, the SBE is a highly efficient cleaning technology for the removal of residual pesticides from tea, and if the appearance of tea is not important in the production process, tea leaves should first be crushed and then extracted using the SBE, in order to wipe out residual pesticides thoroughly.

## Figures and Tables

**Table 1:** The design of Box-Behnken and the response values for bifenthrin extract content.

No.	A (Temperature) (°C)	B (Time) (min)	C (Extraction Cycles)	Bifenthrin	
				*Y* (Extraction Content) (μg/g)	Extraction Efficiency (%)
1	30	30	1	3.01	59
2	45	20	1	4.58	89
3	30	10	3	2.82	55
4	30	30	3	3.23	63
5	15	20	3	3.03	59
6	30	10	1	3.21	63
7	30	20	2	2.91	57
8	15	30	2	2.58	50
9	15	20	1	2.07	40
10	15	10	2	2.54	50
11	30	20	2	3.05	60
12	30	20	2	2.87	56
13	45	30	2	4.67	91
14	45	20	3	3.69	72
15	30	20	2	2.83	55
16	30	20	2	2.76	54
17	45	10	2	4.31	84
18 (control sample *)	-	-	-	5.12	100

* indicated the sample without the butane treated process, but with the spraying of pesticides.

**Table 2:** The analysis of variance (ANOVA) for the experimental results.

Source	Sum of Squares	Df ^1^	Mean Square	*F*-Value	*p*-Value	Significance
Model	8.27	9	0.92	54.53	<0.0001	**
A-temperature	6.18	1	6.18	366.64	<0.0001	**
B-time	0.047	1	0.047	2.76	0.1406	
C-extraction cycles	1.25 × 10^−3^	1	1.25 × 10^−3^	0.074	0.7932	
AB	0.026	1	0.026	1.52	0.2575	
AC	0.86	1	0.86	50.78	0.0002	**
BC	0.093	1	0.093	5.52	0.0511	
A^2^	0.88	1	0.88	52.42	0.0002	**
B^2^	0.14	1	0.14	8.37	0.0232	*
C^2^	1.05 × 10^−6^	1	1.05 × 10^−6^	6.25 × 10^−5^	0.9939	
Residual	0.12	7	1.70 × 10^−2^			
Lack of Fit	0.071	3	2.40 × 10^−2^	2.03	0.2519	
Pure Error	4.70 × 10^−2^	4	1.20 × 10^−2^			
Cor Total	8.39	16				

* indicated the significant difference (*p*-value < 0.05); ** indicated a more significant difference (*p*-value < 0.01); ^1^ Df means the degree of freedom.

**Table 3:** Catechins, theanine and caffeine contents in tea leaves treated by subcritical butane extraction.

Analytes		Linear Regression Equation	*R*^2^	Treated Samples (%)	Control Samples (%)	Loss Rate (%)
**Catechins (six kinds)**	Gallic acid	*Y* = − 13.105*x* + 72.313	0.9940	0.43	0.46	6.52
	(−)-epigallocatechin	*Y* = − 170.28*x* + 902.70	0.9926	2.67	2.86	6.64
	Catechin	*Y* = 155.17*x* − 40.97	0.9963	0.41	0.48	14.58
	(−)-Epigallocatechin gallate	*Y* = 956.38*x* − 9.84	0.9995	4.08	4.20	2.86
	(−)-Epicatechin	*Y* = 220.80*x* − 123.50	0.9928	1.43	1.53	6.53
	(−)-Epicatechin gallate	*Y* = 26.590*x* − 28.09	0.9989	1.68	1.76	4.55
**Theanine**		*Y* = 268.67*x* + 5.75	0.9991	1.47	1.54	4.54
**Caffeine**		*Y* = 44.24*x* − 3.26	0.9978	3.58	3.63	1.37

**Table 4:** Content changes of the key aroma compounds in the intact and the crushed tea leaves treated by SBE method.

Analytes	Relative Content of Intact Samples			Relative Content of Crushed Samples		
	Control Sample (%) ^1^	Treated Sample (%) ^2^	Variation (%) ^3^	Control Sample (%) ^4^	Treated Sample (%) ^5^	Variation (%)
**Linalool**	0.0019	0.0018	3.48%	0.0057	0.0027	52.73%
**Linaloloxide**	0.0201	0.0183	8.73%	0.0427	0.0229	46.44%
**Nerol**	0.0069	0.0063	8.54%	0.0209	0.0084	60.06%
**β-ionone**	0.0748	0.0266	64.49%	0.1847	0.0960	48.04%
**2-(*E*)-hexenal**	0.0079	0.0078	2.22%	0.0213	0.0100	53.05%

^1^ The sample was intact and not processed by SBE; ^2^ the sample was intact and processed by SBE under the optimum condition; ^3^ variation rate = (control sample − treated sample) × 100/control sample; ^4^ the sample was crushed and not processed by SBE; ^5^ the sample was crushed and processed by SBE under the condition numbered 3 as showed in the Table 1.

## Supplementary Materials

Supplementary materials are available online.
