# Supplementary material for: Effective Subcritical Butane Extraction of Bifenthrin Residue in Black Tea
Source: Molecules. 2017 Mar 30;22(4):560. doi: 10.3390/molecules22040560 (PMC6154673; doi:10.3390/molecules22040560)
Supplement: Supplementary file 1 [file molecules-22-00560-s001.pdf]

Table S1. Aroma components of the intact tea leaves treated by SBE technology.

Category	No.	Retention time	Compounds	Molecular formula	Relative mass fraction (%)	
					Control sample	Treated sample
Alcohols	1	8.512	cis-2-Pentenol	$C_5H_{10}O$	0.0279	0.0298
	6	12.04	Cyclohexanol	$C_6H_{12}O$	0.0079	0.0122
	18	18.015	cis-alpha,alpha,5-Trimethyl-5-vinyltetrahydrofuran-2-methanol	$C_{10}H_{18}O_2$	0.0077	0.0066
	19	18.51	Linaloloxide	$C_{10}H_{18}O_2$	0.0201	0.0183
	21	18.826	Linalool	$C_{10}H_{18}O$	0.0019	0.0018
	22	18.945	Hotrienol	$C_{10}H_{16}O$	0.0066	0.0055
	27	23.341	Nerol	$C_{10}H_{18}O$	0.0069	0.0063
	28	24.552	Anethole	$C_{10}H_{12}O$	0.0032	0.0013
Aldehydes	2	9.369	Hexenal	$C_6H_{12}O$	0.0234	0.0251
	4	10.295	Furfural	$C_5H_4O_2$	0.0446	0.0436
	5	10.941	2-(E)-hexenal	$C_6H_{10}O$	0.0079	0.0078
	8	14.372	5-Methyl furfural	$C_6H_6O_2$	0.0284	0.0232
	9	14.524	Benzaldehyde	C_7H_6O	0.0154	0.0137
	13	15.538	2,4-(E,E)-Heptadienal	$C_7H_{10}O$	0.0118	0.0064
	15	16.027	2,4-Heptadienal	$C_7H_{10}O$	0.0433	0.0330
	17	17.162	Benzeneacetaldehyde	C_8H_8O	0.0051	0.0038
	26	22.667	β-Cyclocitral	$C_{10}H_{16}O$	0.0014	0.0012
	29	25.363	E, E-2, 4-Decedienal	$C_{10}H_{16}O$	0.0035	0.0014
Ketones	11	15.071	Methylheptenone	$C_8H_{14}O$	0.0119	0.0084
	20	18.632	3,5-Octadien-2-one	$C_8H_{12}O$	0.0012	0.0008
	24	21.641	1-(2-methylphenyl)- Ethanone	$C_9H_{10}O$	0.0034	0.0025
	31	29.71	β-ionone	$C_{13}H_{20}O$	0.0748	0.0266
	32	29.798	4-(1,2-Oxido-2,6,6-trimethylcyclohexyl)-3-buten-2-one	$C_{13}H_{20}O_2$	0.0304	0.0163
Esters	25	21.869	Methyl salicylate	$C_8H_8O_3$	0.0045	0.0033
	30	29.312	γ-Decalactone	$C_{10}H_{18}O_2$	0.0575	0.0324
	33	31.197	dihydroactinidiolide	$C_{11}H_{16}O_2$	0.0019	0.0010
Terpenes	16	16.71	D-limonene	$C_{10}H_{16}$	0.0012	0.0015

Alkanes	34	42.135	Heneicosane	$C_{21}H_{44}$	0.0685	0.0668
	36	43.616	Docosane	$C_{22}H_{46}$	0.1851	0.1576
	37	45.264	Tricosane	$C_{23}H_{48}$	0.4716	0.4149
	38	47.189	Heptadecane	$C_{27}H_{56}$	0.1484	0.1455
	39	49.516	Octacosane	$C_{28}H_{58}$	0.1469	0.1618
	40	52.403	nonacosane	$C_{29}H_{60}$	0.6607	0.7912
Other compounds	3	10.091	Methylpyrazine	$C_5H_6N_2$	0.0144	0.0125
	7	12.793	2,5-Dimethylpyrazine	$C_6H_8N_2$	0.0191	0.0197
	10	14.781	Hexanoic acid	$C_6H_{12}O_2$	0.0352	0.0217
	12	15.317	2-Pentylfuran	$C_9H_{14}O$	0.0059	0.0071
	14	15.736	2-Ethyl-5- Methylpyrazine	$C_7H_{10}N_2$	0.0075	0.0064
	23	20.117	Benzyl cyanide	C_8H_7N	0.0075	0.0047
	37	42.317	Phytol	$C_{20}H_{40}O$	0.0671	0.1183

Table S2. Aroma components of the crushed tea leaves treated by SBE technology

Category	No.	Retention time	Compounds	Molecular formula	Relative mass fraction (%)	
					Control sample	Treated sample
Alcohols	1	8.519	cis-2-Pentenol	$C_5H_{10}O$	0.0603	0.0569
	6	12.074	Cyclohexanol	$C_6H_{12}O$	0.0145	0.0056
	16	18.075	cis-alpha, alpha, 5-Trimethyl-5-vinyltetrahydrofuran-2-methanol	$C_{10}H_{18}O_2$	0.0168	0.0088
	17	18.567	Linaloloxide	$C_{10}H_{18}O_2$	0.0428	0.0229
	19	18.884	Linalool	$C_{10}H_{18}O$	0.0058	0.0027
	20	19.002	Hotrienol	$C_{10}H_{16}O$	0.0133	0.0091
	25	23.384	Nerol	$C_{10}H_{18}O$	0.021	0.0084
	26	24.567	Anethole	$C_{10}H_{12}O$	0.0096	0.006
Aldehydes	2	9.377	Hexenal	$C_6H_{12}O$	0.0624	0.0425
	4	10.318	Furfural	$C_5H_4O_2$	0.1128	0.0594
	5	10.946	2-(E)-hexenal	$C_6H_{10}O$	0.0214	0.01
	8	14.42	5-Methyl furfural	$C_6H_6O_2$	0.0789	0.0391
	9	14.564	Benzaldehyde	C_7H_6O	0.0335	0.0198
	15	17.208	Benzeneacetaldehyde	C_8H_8O	0.0149	0.0064
	24	22.698	β-Cyclocitral	$C_{10}H_{16}O$	0.0047	0.002
	27	25.375	E, E-2, 4-Decedienal	$C_{10}H_{16}O$	0.0074	0.0013
Ketones	10	15.086	Methylheptenone	$C_8H_{14}O$	0.0257	0.0176
	18	18.666	3,5-Octadien-2-one	$C_8H_{12}O$	0.0026	0.0014
	22	21.665	1-(2-methylphenyl)- Ethanone	$C_9H_{10}O$	0.0079	0.0042
	29	29.792	β-ionone	$C_{13}H_{20}O$	0.185	0.0959
Esters	13	16.562	1-Methyl-2-(1-methylethyl) benzene	$C_{10}H_{14}$	0.0143	0.0063
	23	21.895	Methyl salicylate	$C_8H_8O_3$	0.0109	0.0064
	28	29.448	γ-Decalactone	$C_{10}H_{18}O_2$	0.1814	0.0467

	30	40.109	Dibutyl phthalate	$C_{16}H_{22}O_4$	0.0089	0.0094
Terpenes	14	16.72	D-limonene	$C_{10}H_{16}$	0.0082	0.0046
	31	42.153	Heneicosane	$C_{21}H_{44}$	0.0936	0.0681
	33	43.622	Docosane	$C_{22}H_{46}$	0.0585	0.0544
Alkanes	34	45.273	Tricosane	$C_{23}H_{48}$	0.1881	0.1164
	35	49.538	Octacosane	$C_{28}H_{58}$	0.0725	0.0101
	36	52.428	nonacosane	$C_{29}H_{60}$	0.3479	0.142
	32	42.339	Phytol	$C_{20}H_{40}O$	0.4809	0.0776
	3	10.081	Methylpyrazine	$C_5H_6N_2$	0.0402	0.0177
Other	7	12.814	2,5-Dimethylpyrazine	$C_6H_8N_2$	0.0534	0.0246
compounds	11	15.328	2-Pentylfuran	$C_9H_{14}O$	0.0224	0.0164
	12	15.76	2,5-Dimethylpyrazine	$C_7H_{10}N_2$	0.018	0.0097
	21	20.154	Benzyl cyanide	C_8H_7N	0.0241	0.0094
